# Comparative Genomics and Phylogenomics of East Asian Tulips (*Amana*, Liliaceae)

**DOI:** 10.3389/fpls.2017.00451

**Published:** 2017-04-04

**Authors:** Pan Li, Rui-Sen Lu, Wu-Qin Xu, Tetsuo Ohi-Toma, Min-Qi Cai, Ying-Xiong Qiu, Kenneth M. Cameron, Cheng-Xin Fu

**Affiliations:** ^1^Key Laboratory of Conservation Biology for Endangered Wildlife of the Ministry of Education, and Laboratory of Systematic & Evolutionary Botany and Biodiversity, College of Life Sciences, Zhejiang UniversityHangzhou, China; ^2^Botanical Gardens, Graduate School of Science, University of TokyoTokyo, Japan; ^3^Department of Botany, University of WisconsinMadison, WI, USA

**Keywords:** *Amana*, *Tulipa*, *Erythronium*, Liliaceae, chloroplast genome, genomic structure, phylogenomics

## Abstract

The genus *Amana* Honda (Liliaceae), when it is treated as separate from *Tulipa*, comprises six perennial herbaceous species that are restricted to China, Japan and the Korean Peninsula. Although all six *Amana* species have important medicinal and horticultural uses, studies focused on species identification and molecular phylogenetics are few. Here we report the nucleotide sequences of six complete *Amana* chloroplast (cp) genomes. The cp genomes of *Amana* range from 150,613 bp to 151,136 bp in length, all including a pair of inverted repeats (25,629–25,859 bp) separated by the large single-copy (81,482–82,218 bp) and small single-copy (17,366–17,465 bp) regions. Each cp genome equivalently contains 112 unique genes consisting of 30 transfer RNA genes, four ribosomal RNA genes, and 78 protein coding genes. Gene content, gene order, AT content, and IR/SC boundary structure are nearly identical among all *Amana* cp genomes. However, the relative contraction and expansion of the IR/SC borders among the six *Amana* cp genomes results in length variation among them. Simple sequence repeat (SSR) analyses of these *Amana* cp genomes indicate that the richest SSRs are A/T mononucleotides. The number of repeats among the six *Amana* species varies from 54 (*A. anhuiensis*) to 69 (*Amana kuocangshanica*) with palindromic (28–35) and forward repeats (23–30) as the most common types. Phylogenomic analyses based on these complete cp genomes and 74 common protein-coding genes strongly support the monophyly of the genus, and a sister relationship between *Amana* and *Erythronium*, rather than a shared common ancestor with *Tulipa*. Nine DNA markers (*rps15–ycf1, accD–psaI, petA–psbJ, rpl32–trnL, atpH–atpI, petD–rpoA, trnS–trnG, psbM–trnD*, and *ycf4–cemA*) with number of variable sites greater than 0.9% were identified, and these may be useful for future population genetic and phylogeographic studies of *Amana* species.

## Introduction

Tulips (genus *Tulipa sensu lato*) are among the world's most well-known, beloved, and economically important flowering plants. Their horticultural popularity, especially in Europe during the mid-seventeenth Century, led to bulbs being infamously traded in Holland as a form of speculative currency during a period that came to be known by historians as “tulip mania.” Although, there has been considerable research into the biology of tulips native to the Middle East and North Africa (Eijk et al., 1991; van Tunen et al., 1993; Van Creij et al., 1997; van Rossum et al., 1998; Zonneveld, 2009), much less is known of the East Asian tulips (e.g., *Tulipa edulis*), a group of species that most botanists today recognize as a distinct genus *Amana* Honda (Liliaceae). *Amana* is comprised of ca. six species of geophytic, perennial, understory herbs that are endemic to temperate East Asia (Ohwi and Kitagawa, 1992; Chen and Mordak, 2000; Shen, 2001; Tan et al., 2007; Han et al., 2014). The genus does, indeed, share many morphological characters with *Tulipa* L. (tulips), which is why most taxonomists until recently have classified it within *Tulipa sensu lato* (Sealy, 1957; Mao, 1980; Ohwi and Kitagawa, 1992; Liang, 1995; Tamura, 1998; Shen, 2001). However, *Amana* differs from *Tulipa* sensu stricto in having 2–3(–4) opposite or verticillate bracts in the upper part of the flowering stem and a longer style that is as long as the ovary (Tan et al., 2005). In many features it also resembles the genus *Erythronium* L. (trout lilies) from North America and Eurasia. At present, *Amana* is generally accepted as a separate genus (Tan et al., 2005, 2007; Christenhusz et al., 2013; Han et al., 2014). Recent molecular phylogenetic studies based on a few plastid regions and nuclear ribosomal ITS sequences (Hayashi and Kawano, 2000; Allen et al., 2003; Rønsted et al., 2005; Zarrei et al., 2009; Clennett et al., 2012; Christenhusz et al., 2013; Kim et al., 2013) have generally supported this separation. *Amana, Erythronium*, and *Tulipa* were strongly supported to be a monophyletic group in all of these studies, but the precise sister relationships among them has remained controversial. For example, some studies clustered *Amana* and *Tulipa* together (Hayashi and Kawano, 2000; Zarrei et al., 2009, ITS), whereas others supported a sister relationship between *Erythronium* and *Tulipa* (Allen et al., 2003; Christenhusz et al., 2013). Still others found that *Amana* is most closely related to *Erythronium* (Rønsted et al., 2005; Zarrei et al., 2009, five plastid regions combined; Clennett et al., 2012; Kim et al., 2013). All previous studies appear to have been based on insufficient information and thus could not fully resolve the phylogenetic relationships among these taxa.

**Figure 1:**
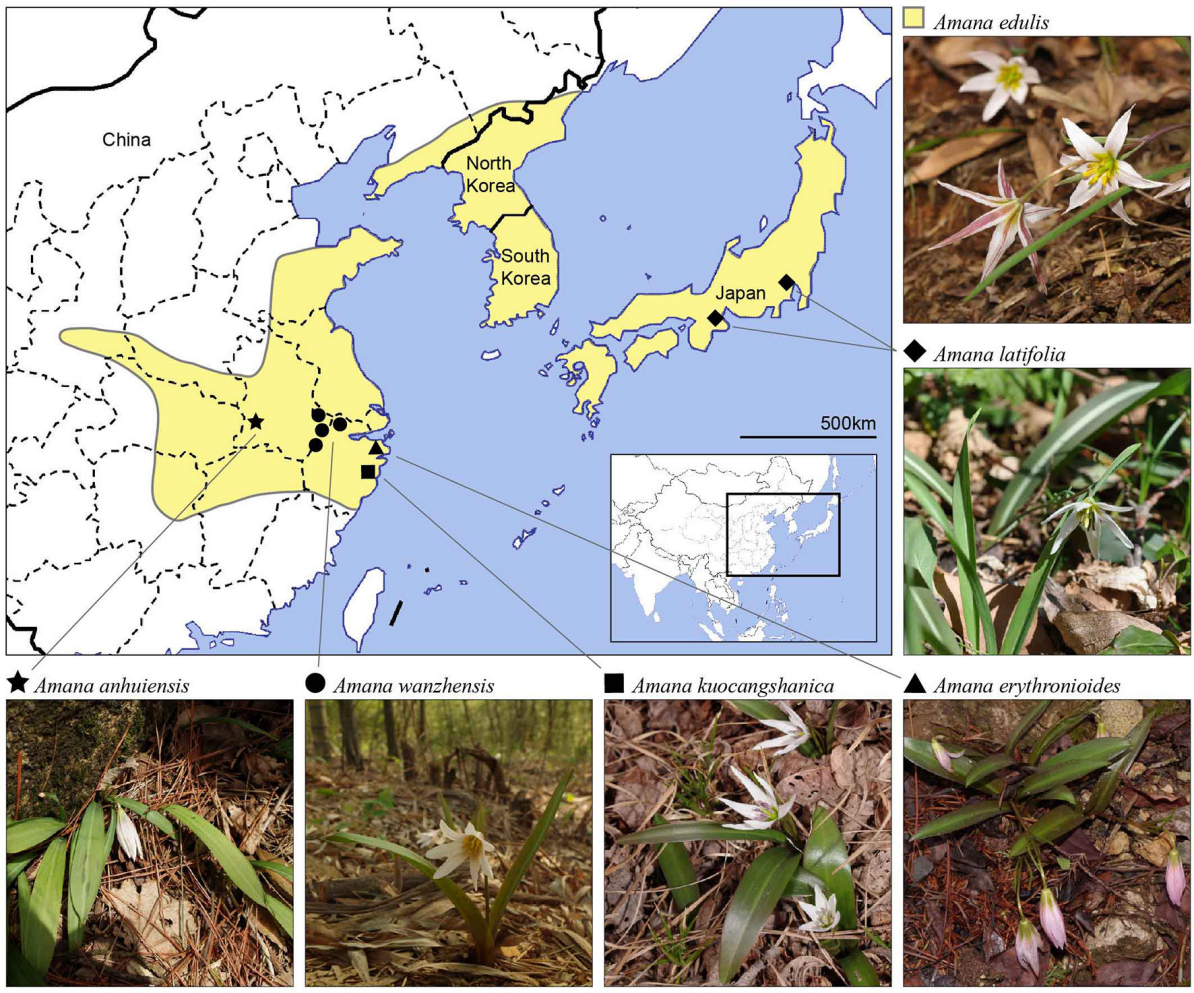
**Distribution map of all six currently recognized ***Amana*** species**. One of the species, *A. edulis*, is widespread and found primarily at low elevations (yellow-shaded areas). The other five species are narrow endemics restricted to disjunct montane habitats (filled symbols).

The six currently recognized species of *Amana* occur in temperate deciduous or subtropical evergreen broad-leaved/mixed forests (Table 1). Within the genus, *Amana edulis* (Miq.) Honda is the most common and widely distributed species, ranging from China (central, eastern and northeastern provinces) to Japan (Honshu, Kyushu, and Shikoku) and the Korean peninsula (Ohwi and Kitagawa, 1992; Chen and Mordak, 2000; Park, 2007). The other five species are narrow endemics with non-overlapping areas among them, but all are broadly sympatric with *A. edulis* (Figure 1). However, these narrow endemic species rarely co-occur with the widespread *A. edulis* in intermixed populations due to the different altitudes of their natural habitats (*A. edulis*: 0–400 m, rarely to 850 m; other species: 600–1,400 m). Specifically, *A. latifolia* (Makino) Honda is restricted to a few sites in Honshu, Japan (Ohwi and Kitagawa, 1992); *A. erythronioides* (Baker) D. Y. Tan and D. Y. Hong and *A. kuocangshanica* D. Y. Tan and D. Y. Hong are confined to a few mountains near the coast of the East China Sea (Chen and Mordak, 2000; Tan et al., 2007), whereas *A. anhuiensis* (X. S. Shen) D. Y. Tan and D. Y. Hong and *A. wanzhensis* L. Q. Huang, B. X. Han and K. Zhang have more interior distributions in eastern China (Shen, 2001; Tan et al., 2008; Han et al., 2014; P. Li, pers. obs.). Despite the taxonomic recognition of six *Amana* species, the evolutionary history and interspecific relationships in this genus are still unclear because most species of *Amana* were missing from previous studies.

**Table 1:** **The basic characteristics of six ***Amana*** species chloroplast (cp) genomes**.

**Characteristics**	***A. edulis***	***A. latifolia***	***A. erythronioides***	***A. anhuiensis***	***A. kuocangshanica***	***A. wanzhensis***
Location	China: Zhejiang	Japan: Tokyo	China: Zhejiang	China: Anhui	China: Zhejiang	China: Anhui
Latitude (N°)	30.2558	35.7183	29.7319	30.7408	28.8058	30.3486
Longitude (E°)	120.1211	139.7464	121.0861	116.4525	120.9131	119.2294
GenBank numbers	KY401425	KY401424	KY401421	KY401423	KY401426	KY401422
Total clean reads	12,248,447	12,552,899	16,581,300	28,408,624	16,591,142	28,290,288
Number of contigs	14,158	10,585	30,992	70,038	30,741	67,996
Contigs used for constructing cp genome	5	6	6	3	3	3
N50 of contigs (bp)	348	342	345	340	338	334
Cp genome coverage (×)	376.3	307.4	123.2	378.2	171.7	452.1
Total cpDNA Size (bp)	151,136	150,613	150,858	150,842	151,058	150,913
LSC length (bp)	82,029	81,482	82,218	82,119	81,916	81,758
SSC length (bp)	17,429	17,439	17,366	17,465	17,424	17,445
IR length (bp)	25,839	25,846	25,637	25,629	25,859	25,855
Total GC content (%)	36.7	36.8	36.7	36.7	36.7	36.7
LSC	34.6	34.7	34.6	34.6	34.6	34.7
SSC	30.2	30.0	30.0	30.0	30.0	30.0
IR	42.3	42.3	42.4	42.4	42.3	42.3
Total number of genes	132	132	132	132	132	132
Protein-coding genes	78	78	78	78	78	78
rRNAs genes	4	4	4	4	4	4
tRNAs genes	30	30	30	30	30	30
Duplicated genes	20	20	20	20	20	20

Not only are these plants valuable to humans as ornamentals, but they have considerable ethnobotanical uses as well. The bulb of *Amana edulis* is edible and commonly used as herbal medicine or starch source in China (Chen and Mordak, 2000). It has been used in traditional Chinese medicine (TCM) under the common name “Guangcigu” to treat sore throats, scrofula, ulcers and postpartum blood stasis (Chinese Herbalism Editorial Board, 1999). Other species in the genus *Amana* can be found as adulterants of Guangcigu, and these may result in different pharmacological actions, but such adulterants are often misidentified due to the similarity in their appearance with *A. edulis* (Ma H. L. et al., 2014). The increasing demand for wild-collected material of these economically important plants has brought about overexploitation of the natural populations in some regions. Therefore, a rapid and accurate method for species identification of *Amana* species is needed not only to facilitate proper medicinal uses, but also to aid conservation management.

In this study, we chose to analyze the complete chloroplast (cp) genomes of all six *Amana* species because of the plastome's conservative rate of evolution, absence of recombination, uniparental inheritance, and small effective population size (Birky et al., 1983). These are the same reasons that cpDNA sequences have been extensively used in studies of plant population genetics, phylogeography, phylogeny, and DNA barcoding (Jansen et al., 2007; Moore et al., 2010; Shaw et al., 2014). Compared with phylogenetic studies limited to a few cpDNA regions, cp phylogenomic studies involve many more informative sites for potentially greater resolution and support (Burke et al., 2012). With the rapid development of next-generation sequencing, cp genome-scale data have been increasingly employed to infer phylogenetic relationships at almost any taxonomic levels in the past decade (Jansen et al., 2007; Moore et al., 2007, 2010; Parks et al., 2009; Barrett et al., 2013; Ma P. F. et al., 2014; Carbonell-Caballero et al., 2015; Zhang et al., 2016). In addition, based on comparative genomic analyses, cp genomic hotspots can be identified as DNA barcodes in discriminating species, in terms of informative regions for a specific plant genus, tribe or family (Doorduin et al., 2011; Li et al., 2013, 2014). Our objectives are to: (1) characterize and compare the cp genomes of all six *Amana* in order to gain insights into their evolutionary patterns; (2) resolve the phylogenetic relationships among all *Amana* species and among closely related genera; (3) screen and identify the most rapidly evolving DNA regions of the *Amana* genome for species identification and future phylogeographic studies of the genus.

## Materials and methods

### Plant samples, DNA extraction and sequencing

Fresh leaf samples of six *Amana* species, five from China and one from Japan (Table 1), were field-collected and dried with silica gel. Voucher herbarium specimens were deposited at the Herbarium of Zhejiang University (HZU). We extracted total DNA from ca. 3 mg of the silica-gel dried leaf tissue for each species using DNA Plantzol Reagent (Invitrogen) and following the manufacturer's protocol. The qualities and quantities of genomic DNA were checked on an Agilent BioAnalyzer 2100 (Agilent Technologies). Short-insert (500 bp) paired-end libraries were generated by using Genomic DNA Sample Prep Kit (Illumina) according to the manufacturer's protocol. Genomic DNA of each species was indexed by barcode tags and then pooled together for sequencing in one lane of HiSeq™ 2500 (Illumina, San Diego, California, USA) at Beijing Genomics Institute (BGI, Shenzhen, China).

### Genome assembly and annotation

For each *Amana* species, raw reads (125 bp read length) were firstly cleaned by removing low-quality reads with Phred scores of <20 using the CLC-quality trim tool (http://www.clcbio.com/products/clc-assembly-cell/). Secondly, we assembled the clean reads into contigs on the CLC *de novo* assembler (http://www.clcbio.com/products/clc-assembly-cell/), under the following settings: minimum contig length of 200 bp, mismatch cost of 2, deletion and insertion costs of 3, length fraction of 0.8, and similarity fraction of 0.8. Thirdly, all the contigs were aligned to the reference genome (*Lilium longiflorum* Thunb., KC968977) using BLAST (http://blast.ncbi.nlm.nih.gov/), and aligned contigs were oriented according to the reference genome. Then, contigs were aligned with the reference genome for constructing the draft chloroplast genome of each *Amana* species in GENEIOUS V9.0.5 (http://www.geneious.com). Finally, clean reads were re-mapped to the draft genome and yielded the complete chloroplast genome sequences.

Preliminary annotation of these *Amana* chloroplast genomes was conducted on the program Dual Organellar GenoMe Annotator (DOGMA; Wyman et al., 2004). DOGMA annotations were further corrected for the start/stop codons and intron/exon boundaries by comparison with homologous genes from *L. longiflorum* (GenBank accession no. KC968977) and *Fritillaria hupehensis* (GenBank accession no. KF712486) using MAFFT v7 (Katoh and Standley, 2013). In addition, tRNAscan-SE (Schattner et al., 2005) was used to verify the tRNA genes with default parameters, and the ultimately annotated chloroplast genomes were deposited in GenBank (accession numbers listed in Table 1). The cp genome maps were drawn in OrganellarGenome DRAW (Lohse et al., 2007). Codon usage, as well as relative synonymous codon usage (RSCU, Sharp and Li, 1987) value was estimated for all exons of protein-coding genes with the program CODONW V1.4.2 (http://codonw.sourceforge.net/).

### Genome comparative analysis and molecular marker identification

Chloroplast genome comparisons across the six *Amana* species was performed in Shuffle-LAGAN mode on the mVISTA program (genome.lbl.gov/vista/index. shtml, Frazer et al., 2004), using the annotation of *A. kuocangshanica* as a reference. To evaluate whether different cp genome regions underwent different evolution patterns in this genus, and to explore highly variable regions for future population genetic and species identification studies, we sequentially extracted both coding regions and noncoding regions (including intergenic spacers and introns) after alignment using MAFFT v7 under the two criteria that aligned length is >200 bp and at least one mutation site is present. After that, the nucleotide variability of these regions was evaluated with DNASP V5.10 (Librado and Rozas, 2009).

### Identification of repeat sequences and simple sequence repeats

REPUTER (Kurtz and Schleiermacher, 1999) was used to determine the size and position of repeat sequences, which included direct, inverted, complement and reverse repeats in the *Amana* chloroplast genomes. The minimum length of repeat size and sequence identity was set to 30 bp and >90%. MISA perl script (Thiel et al., 2003) was applied to detect the simple sequence repeats (SSRs) in the six *Amana* cp genomes with thresholds of 10, 5, 4, 3, 3, 3 repeat units for mono-, di-, tri-, tetra-, penta-, and hexanucleotide SSRs, respectively.

### Phylogenetic analyses

Phylogenetic analyses were conducted on the six *Amana* species and one species each for *Erythronium, Tulipa* and *Lloydia*, using *L. longiflorum* (KC968977) and *Fritillaria cirrhosa* D. Don (KF769143) as outgroups based on previous studies (Rønsted et al., 2005; Kim et al., 2013). Chloroplast sequences of these 11 species were aligned using MAFFT v7. In order to evaluate possible alternative hypotheses of phylogeny, topologies were constructed by both maximum likelihood (ML) and Bayesian inference (BI) methods using not only the complete cp genome sequences (162,505 bp), but also the exons of protein-coding genes (78,815 bp). We also tried two different partitioning strategies for the second dataset: (1) separating each gene as a partition, (2) divided the data matrix into three partitions, corresponding to the first, second and third codon positions.

The best-fitting models of nucleotide substitutions were determined by the Akaike Information Criterion (AIC) in JMODELTEST V2.1.4 (Posada, 2008). The GTR+I+G model was most suitable for both datasets. Maximum likelihood analyses were conducted using RAXML-HPC v8.2.8 (Stamatakis, 2014) with 1000 bootstrap replicates at the CIPRES Science Gateway website (Miller et al., 2010). Bayesian inference (BI) analyses were performed in MRBAYES v3.2 (Ronquist and Huelsenbeck, 2003). Two independent Markov Chain Monte Carlo chains were calculated simultaneously for five million generations with trees sampled every 500 generations. The first 25% of calculated trees were discarded as burn-in, and a consensus tree was constructed from the remaining trees to estimate posterior probabilities (PPs).

## Results

### Genome assembly and structural features

After filtering the low-quality reads and adaptor sequences, 12,248,447–28,408,770 clean reads (of 125 bp length) were obtained for the six *Amana* species. Through *de novo* assembly, 10,585 contigs (*A. latifolia*) to 70,038 contigs (*A. anhuiensis*) were assembled with N50 contigs varing from 338 to 348 bp (Table 1). Subsequently, three to six initial contigs which were found to be significantly homologous to the reference genome were combined to generate each chloroplast genome, with no gaps or missing nucleotides (Ns) found.

**Figure 2:**
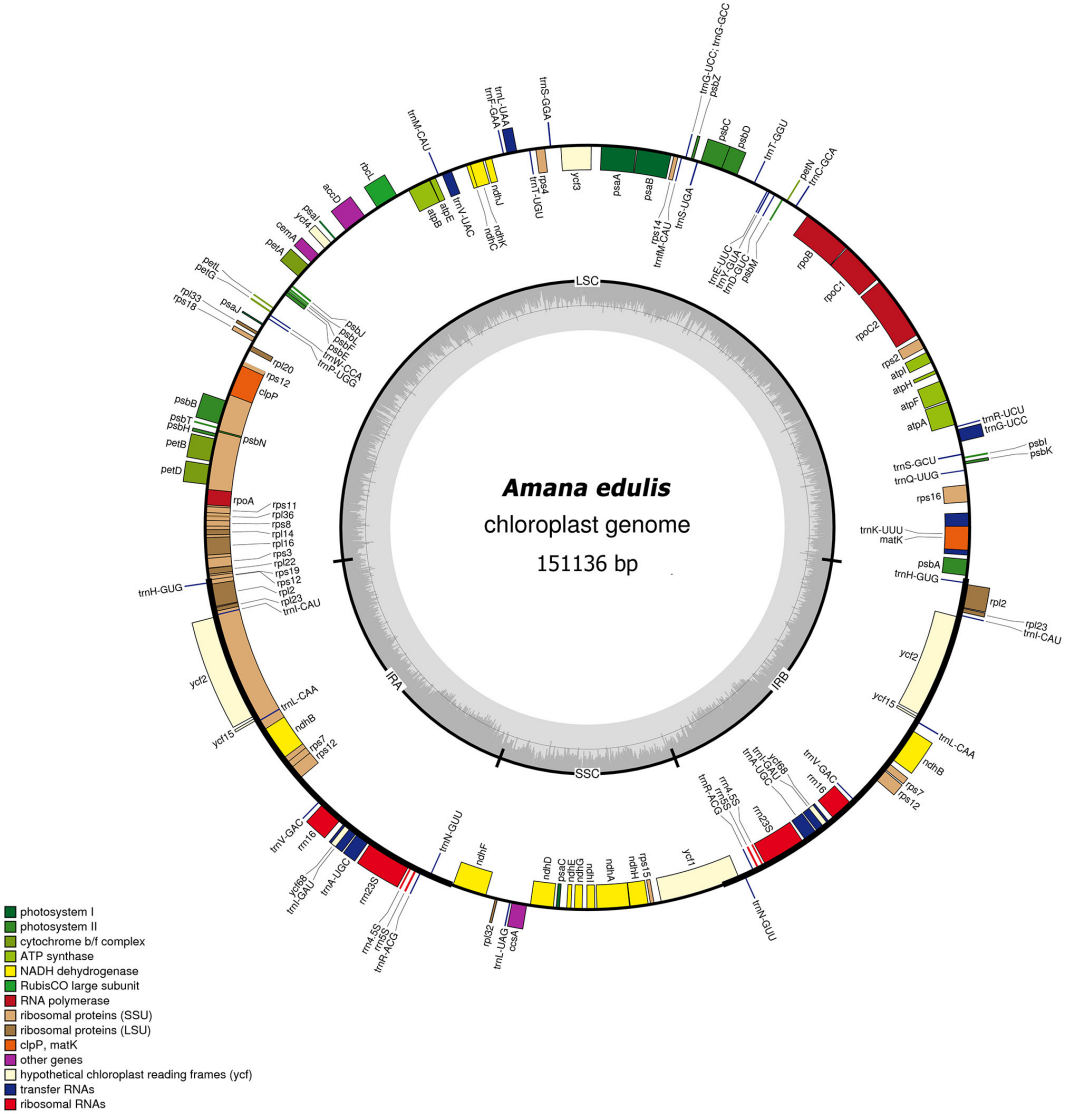
**Gene map of the ***Amana edulis*** chloroplast genome**. Genes shown on the outside of the circle are transcribed clockwise, and genes inside are transcribed counter-clockwise. Genes belonging to different functional groups are color-coded. The darker gray in the inner corresponds to the GC content, and the lighter gray to the AT content. The cp genomes of other five *Amana* species are slightly different with that of *A. edulis* in nucleotide composition, but do not vary in terms of gene content or order.

The full length of the six *Amana* chloroplast genomes ranged from 150,613 to 151,136 bp (Table 1; Figure 2). Akin to other angiosperms, chloroplast genomes of the six *Amana* species present a typical quadripartite structure, including a pair of inverted repeat regions (IR with 25,629–25,859 bp) separated by one large single-copy region (LSC with 81,482–82,218 bp) and one small single-copy region (SSC with 17,366–17,465 bp). All the complete cp genomes with annotation were deposited in GenBank (accession numbers listed in Table 1). The GC content in the LSC, SSC, and IR regions, and also in the whole genome sequences, were nearly identical among the six *Amana* species (Table 1).

The six *Amana* chloroplast genomes contained the same 132 genes, of which 20 were duplicated in the IR regions and 112 were unique genes comprising four rRNA genes, 30 tRNA genes and 78 protein-coding genes (Table 2). Of the 112 distinct genes, 15 held a single intron (nine protein-coding genes and six tRNA genes) and three (*ycf3, clpP*, and *rps12*) possessed two introns. The gene *infA* was lost in all six *Amana* species. The gene *rps12* was trans-spliced; the exon at the 5′ end was located in the LSC region, however the 3′ exon and intron were located in the IR regions. The regions *ycf15, ycf68*, and *ycf1* were identified as pseudogenes because they contained several internal stop codons. Besides, the *rps19* gene located in the IRa/LSC junction region lost their protein-coding ability because of incomplete gene duplication. The similar event was also observed in the *ycf1* region at the IRb and SSC border (see below for detailed information).

**Table 2:** **Gene composition in six ***Amana*** chloroplast genomes**.

**Groups of gene**	**Name of gene**
Ribosomal RNAs	rrn16 (×2), rrn23 (×2), rrn4.5 (×2), rrn5 (×2)
	trnK-UUU^a^, trnQ-UUG, trnS-GCU, trnG-GCC^a^, trnR-UCU
	trnC-GCA, trnD-GUC, trnY-GUA, trnE-UUC, trnT-GGU
	trnS-UGA, trnG-UCC, trnfM-CAU, trnS-GGA, trnT-UGU
Transfer RNAs	trnL-UAA^a^, trnF-GAA, trnV-UAC^a^, trnM-CAU, trnW-CCA
	trnP-UGG, trnH-GUG (×2), trnI-CAU (×2), trnL-CAA (×2)
	trnV-GAC (×2), trnI-GAU^a^ (×2), trnA-UGC^a^ (×2), trnR-ACG (×2)
	trnN-GUU (×2), trnL-UAG
Photosystem I	psaB, psaA, psaI, psaJ, psaC
Photosystem II	psbA, psbK, psbI, psbM, psbD, psbC, psbZ, psbJ, psbL, psbF, psbE, psbB, psbT, psbN, psbH
Cytochrome	petN, petA, petL, petG, petB^a^, petD^a^
ATP synthase	atpA, atpF^a^, atpH, atpI, atpE, atpB
Rubisco	rbcL
NADH dehydrogenease	ndhJ, ndhK, ndhC, ndhB^a^ (×2), ndhF, ndhD, ndhE
	ndhG, ndhI, ndhA^a^, ndhH
ATP-dependent protease subunit P	clpP^b^
Chloroplast envelope membrane protein	cemA
Large units	rpl33, rpl20, rpl36, rpl14, rpl16^a^, rpl22, rpl2^a^ (×2), rpl23 (×2), rpl32
Small units	rps16^a^, rps2, rps14, rps4, rps18, rps12^b^ (×2), rps11, rps8, rps3, rps19, rps7 (×2), rps15
RNA polymerase	rpoC2, rpoC1^a^, rpoB, rpoA
Miscellaneous proteins	matK, accD, ccsA
Hypothetical proteins & Conserved reading frame	ycf3^b^, ycf4, ycf2 (×2), ^Ψ^ycf15 (×2), ^Ψ^ycf68 (×2), ycf1

a *Indicates the genes containing a single intron*.

b *Indicates the genes containing two introns*.

*(×2) indicates genes duplicated in the IR regions; pseudogene was represented by ^Ψ^*.

The number of codons encoded by 78 protein-coding genes in the six *Amana* chloroplast genomes ranged from 24,349 to 24,380. *Amana kuocangshanica* was randomly selected as an example for detailed investigation, owing to the similar result of codon usage and RSCU values for these six species (Table S1). Among the 24,369 codons in *A. kuocangshanica*, 2,529 (10.38%) encoded leucine and 286 (1.17%) encode cysteine, which were the most and least frequent amino acids, respectively (Table S1, Figure S1). In synonymous codons, RSCU value increased with the number of the codons. Furthermore, the RSCU values of 30 codons were greater than 1, suggesting that they are biased codons in the *A. kuocangshanica* chloroplast protein-coding genes (Table S1, Figure S1).

### Boundaries between IR and SC regions

**Figure 3:**
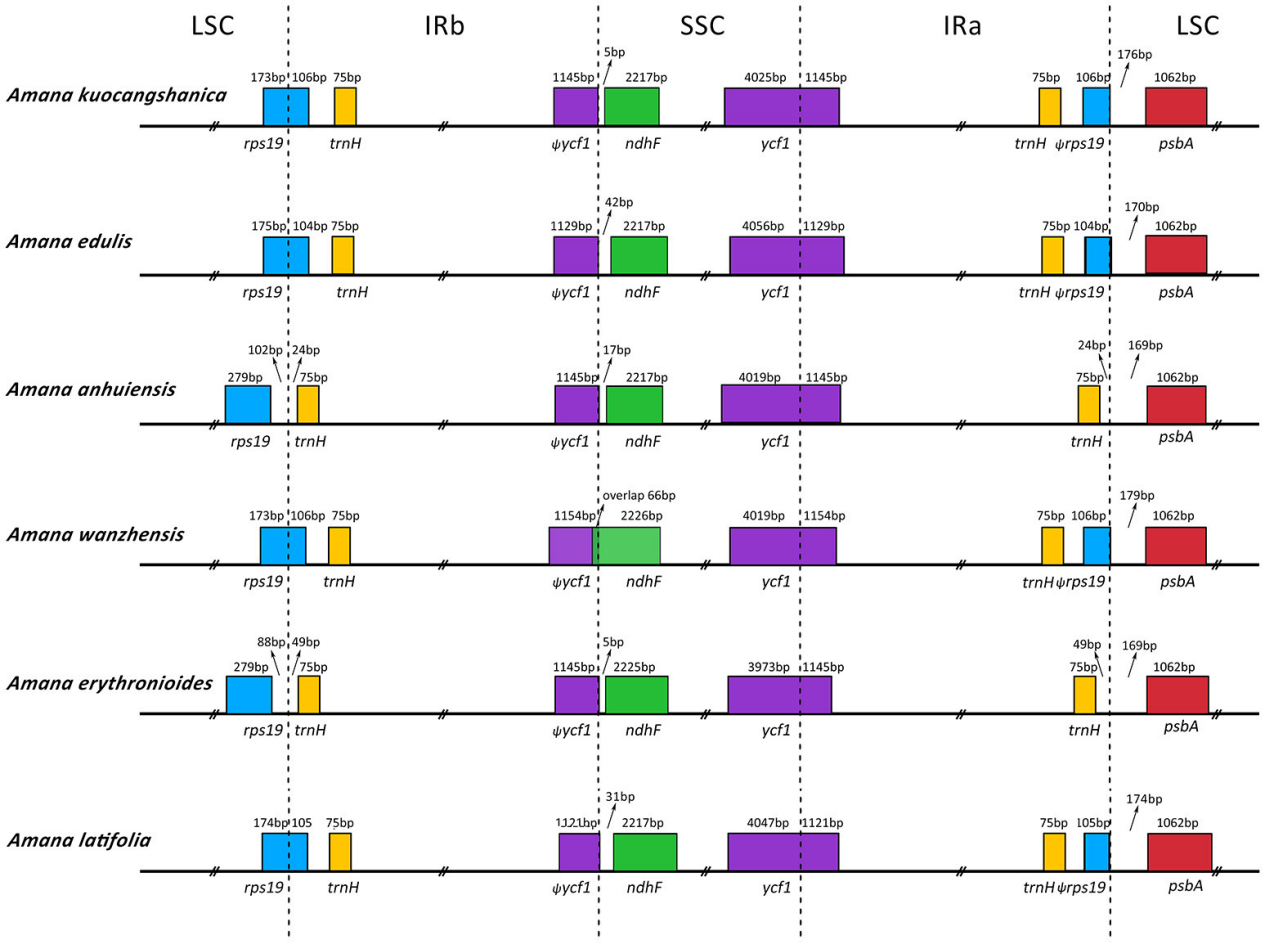
**Comparison of the LSC, IR, and SSC junction positions among six ***Amana*** chloroplast genomes**.

The IR/SC borders with full annotations for adjacent genes were compared among the six *Amana* chloroplast genomes (Figure 3). Except for *A. anhuiensis* and *A. erythronioides*, all the IRb regions expanded by 104–106 bp toward the *rps19* gene with corresponding pseudogene fragment ψ*rps19* created at the IRa/LSC border. Long ψ*ycf1* fragment with 1,121–1,154 bp was located at the IRb regions because the border between SSC and IRa extended into the *ycf1* genes. In addition, the *ndhF* gene in *A. wanzhensis* overlapped with the IRa/SSC border by 66 bp. However, for the other five *Amana* species, the distance between ψ*ycf1* and *ndhF* varied from 5 to 42 bp (Figure 3).

### Comparative genomic analysis and divergence hotspot regions

**Figure 4:**
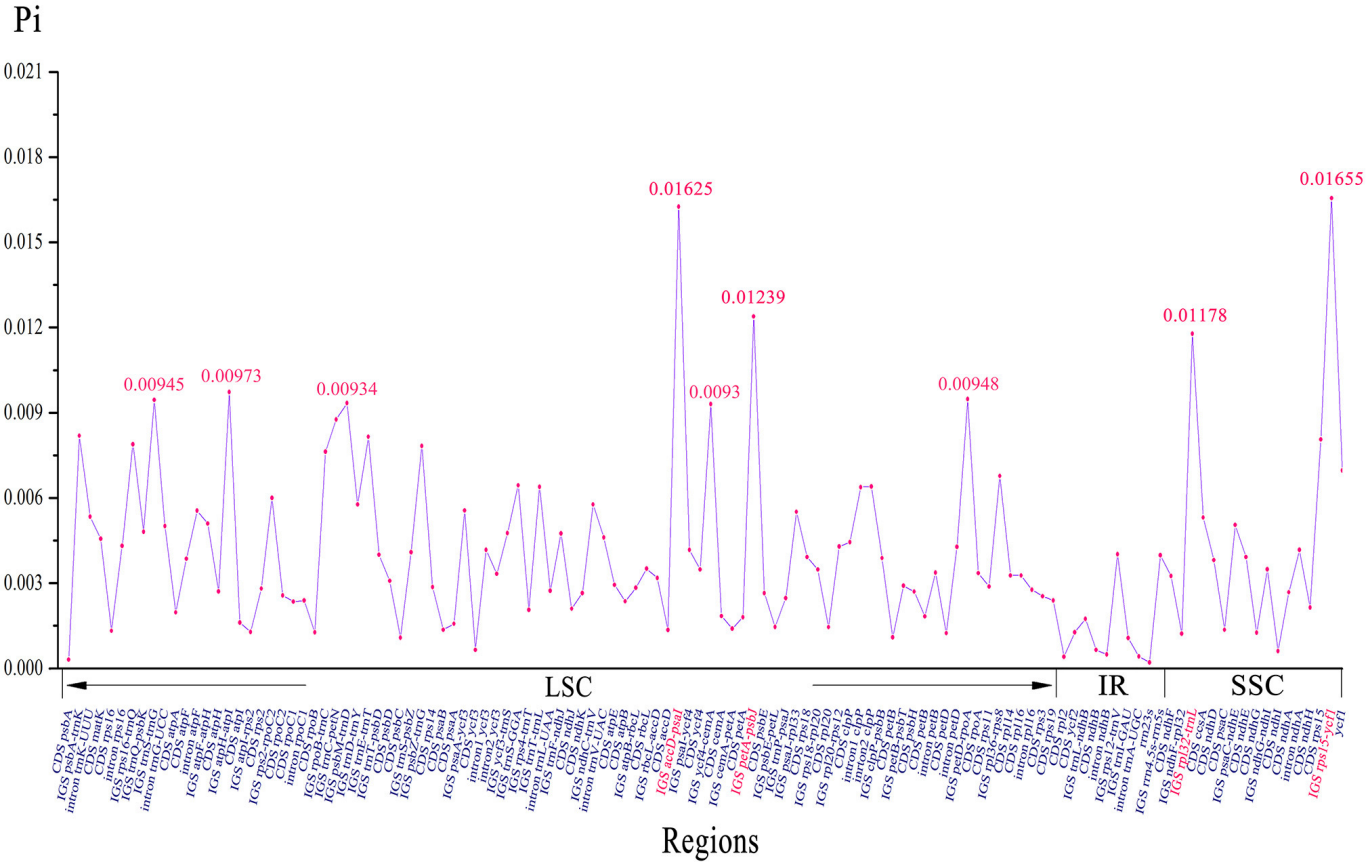
**Nucleotide variability (Pi) values of six ***Amana*** chloroplast genomes**.

We analyzed the comprehensive sequence divergence of the six *Amana* cp genomes using the mVISTA software with the annotation of *A. kuocangshanica* as a reference. A genome-wide alignment revealed globally high sequence similarity (>90% identity) among them (Figure S2). Inverted repeat regions show a lower level of sequence divergence than LSC and SSC regions. In addition, 120 regions were eventually extracted to calculate the nucleotide variability, and the Pi value ranged from 0.02% (*rrn23s*) to 1.66% (*rps15–ycf1*). A total of nine regions (*rps15–ycf1, accD–psaI, petA–psbJ, rpl32–trnL, atpH–atpI, petD–rpoA, trnS–trnG, psbM–trnD*, and *ycf4–cemA*) with a nucleotide diversity >0.9% were recognized as hotspot regions that could be developed as molecular markers for future phylogenetic analysis and plant identification studies (Figure 4, Table S2).

### Repeat analysis and SSR polymorphisms

A total of 371 repeats including 161 forward, 195 palindromic and 15 reverse repeats were identified in the six *Amana* cp genomes using the REPUTER software. *Amana kuocangshanica* possessed the greatest total number of repeats (69), while *A. anhuiensis* contained the fewest (54) (Figure S3A). For each *Amana* species, the majority of repeats (62.3% in *A. kuocangshanica* – 76.2% in *A. edulis*) ranged in size between 30 and 40 bp (Figure S3B). Repeats located in homologous regions with the same lengths were identified as shared repeats. Under this criterion, 38 repeats were shared by all *Amana* species and seven repeats were shared by five of the *Amana* species (all except *A. edulis*). *Amana edulis* showed the most distinct repeats (19), whereas *A. anhuiensis* had the least number (2) (Figure S3C, Table S3).

Each *Amana* species contained 69 (*A. latifolia*) to 76 (*A. wanzhensis*) SSRs, and more than half were composed of A or T bases (Figure S4A, Table S4). In the total 438 SSR regions, the proportion of the repeats situated in the intergenic spacer (IGS) regions led to 58.68%, while the regions located in the coding DNA sequence (CDS), CDS introns, tRNA introns and ψ*ycf1* accounted for 15.53, 14.38, 4.34, and 7.08%, respectively (Figure S4B, Table S4). In addition, 29 SSRs (excluding mononucleotide SSRs) were identified as polymorphic SSRs between *Amana* species (Table S5), which could be useful for further population studies. Three criteria for identification were followed: (1) SSRs possessed the same repeat units (2) the number of repeat units is different and (3) SSRs located in the homologous regions.

### Phylogenetic analyses

**Figure 5:**
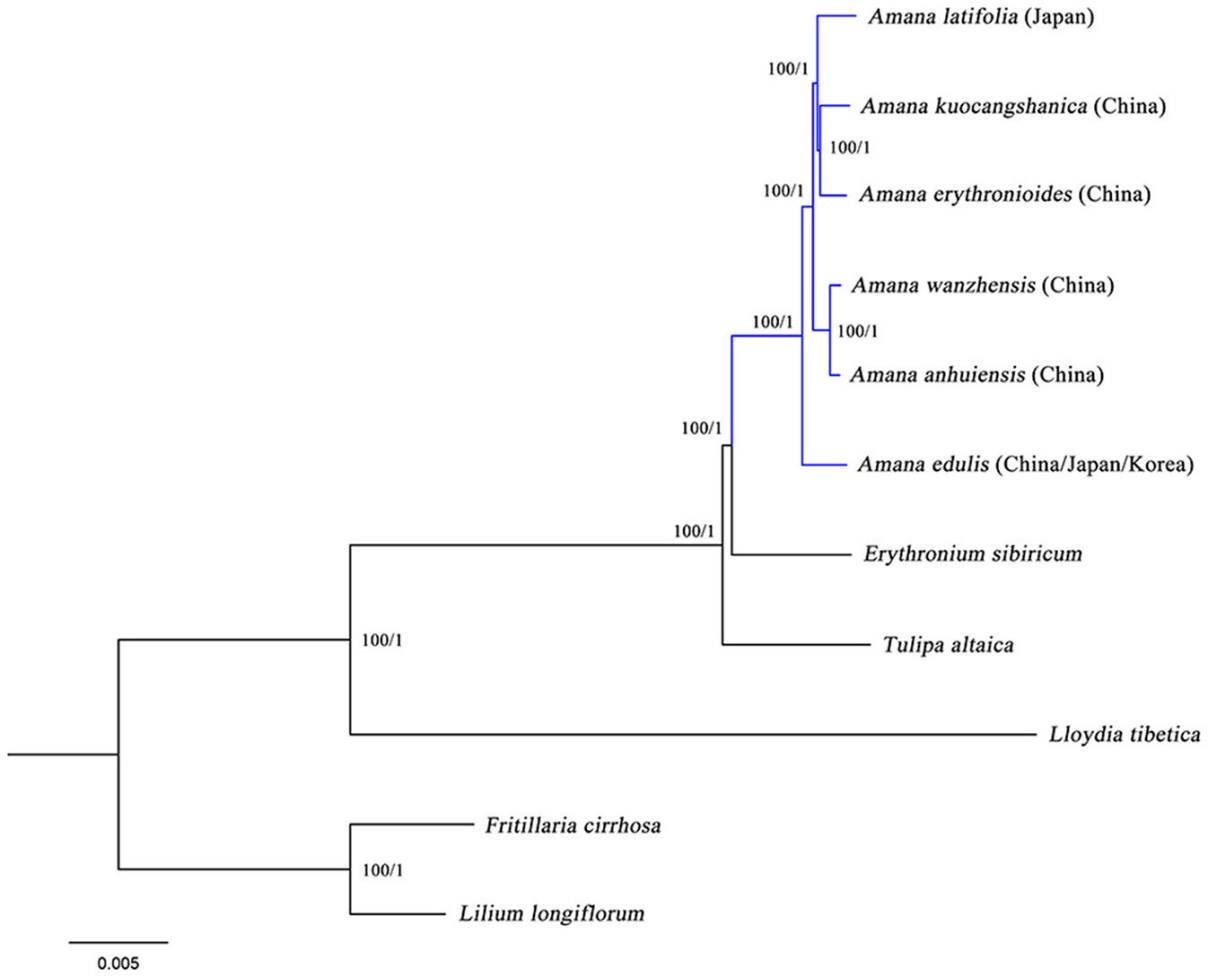
**Phylogenetic relationships among ***Amana***, ***Erythronium***, ***Tulipa*** and within ***Amana*** inferred from maximum likelihood (ML) and Bayesian inference (BI) based on complete genome sequences (162,505 bp)**. Numbers above the lines represent ML bootstrap values and BI posterior probabilities. A phylogenetic tree resulting from analysis of 74 protein-coding genes (78,815 bp) was fully congruent with this topology.

In the present study, two datasets (whole chloroplast genome sequences and shared protein-coding genes) from the six *Amana* species, together with *Erythronium sibiricum, Tulipa altaica, Lloydia tibetica* and two outgroups, were used to conduct various phylogenetic analyses. Both ML and BI methods, based on different datasets and partitioning strategies, produced highly congruent topologies (Figure 5). The phylogenetic trees based on complete genome sequences, which had full support at every nodes [ML bootstrap (BS) = 100%, Bayesian posterior probabilities (PP) = 1], are shown here. *Amana, Erythronium*, and *Tulipa* were fully supported as a monophyletic group, in which the *Amana* species were resolved as a monophyletic clade that was sister to *E. sibiricum*. For the six *Amana* species, *A. edulis* was sister to a clade of the other remaining species with (*A. erythronioides* + *A. kuocangshanica*) and *A. latifolia* sharing a common ancestor and sister to (*A. anhuiensis* + *A. wanzhensis*) (Figure 5).

## Discussion

### Comparative genomics

Our results revealed that the overall gene content and arrangement within the six *Amana* cp genomes are largely similar. This is expected considering the morphological similarities and presumed recent age of divergence among them. At the same time, however, we have been able to document that the plastomes of these species do, indeed, vary, even if their differences are small. The IR/LSC boundaries in the *Amana* chloroplast genomes (except those of *A. erythronioides* and *A. anhuiensis*) expand into the *rps*19 gene. This is congruent with a typical monocot cp genome structure (Yang et al., 2013). Among other taxa in the family Liliaceae, IR expansion into *rps19* has been observed in *Lilium* (Kim and Kim, 2013), *Fritillaria* (Li et al., 2014), and *Cardiocrinum* (Lu et al., 2016). This suggests that the expansion of the IR/LSC junctions into *rps19* may be an ancestral symplesiomorphy of the family Liliaceae, and thus provides no relevant phylogenetic information for addressing intrafamilial questions.

Millen et al. (2001) suggested that *infA*, which codes for translation initiation factor 1, has been entirely lost or has become a pseudogene approximately 24 separate times in 309 angiosperms. According to their results, the parallel loss of *infA* from the chloroplast genome occurred in both *Tricyrtis* (Liliaceae) and *Smilax* (Smilacaceae), which are members of Liliales. Kim and Kim (2013) revealed that this event also occurred in *Alstroemeria* (Alstroemeriaceae), which is closer to the basal Liliales than *Smilax*. Besides, they found that *infA* existed but seemed to have lost its function in the *Lilium* (Liliaceae) cp genome, because it had AAT instead of ATG in the start codon position and includes two premature stop codons. The pseudogenization of *infA* was also found in *Fritillaria* (Li et al., 2014) and *Cardiocrinum* (Lu et al., 2016), close relatives of *Lilium*. However, our study indicates the loss of *infA* in *Amana*. Overall, it shows that *infA* may have been lost in the most recent common ancestor (MRCA) of Liliales, and the pseudogenization of *infA* seems to be a synapomorphy of the (*Lilium* + *Fritillaria*) + *Cardiocrinum* clade. Further study is needed to improve our understanding of *infA* gene evolution in Liliaceae.

### Sister relationship of *Amana* and *Erythronium*

Phylogenetic analyses of the complete chloroplast genome sequences and separate analyses restricted only to 74 common plastid protein-coding genes both produced a well-resolved phylogenetic tree (Figure 5). The close relationship and relatively short branches among *Amana, Erythronium*, and *Tulipa* was confirmed, which is congruent with previous studies (Hayashi and Kawano, 2000; Allen et al., 2003; Rønsted et al., 2005; Zarrei et al., 2009; Clennett et al., 2012; Kim et al., 2013; Petersen et al., 2013). However, our phylogenetic trees unambiguously revealed a sister relationship between *Amana* and *Erythronium* (BS = 100%, PP = 1), clearly supporting the separation of *Amana* from *Tulipa*, as others have argued (Rønsted et al., 2005; Zarrei et al., 2009; Clennett et al., 2012; Kim et al., 2013). Although a closer relationship between *Amana* and *Tulipa*, or of *Erythronium* and *Tulipa*, has been suggested by some former studies, most of them used only a single locus and/or found only weak to medium support for their topologies (rbcL: Hayashi and Kawano, 2000; matK: Allen et al., 2003; ITS: Zarrei et al., 2009). In at least one case (Christenhusz et al., 2013), although a multi-locus phylogenetic analysis of these three genera showed *Erythronium* sister to *Tulipa* rather than to *Amana*, no external outgroup taxa from the sister clade were used to orient the tree, and so that the relative branching order of *Amana, Erythronium*, and *Tulipa* was actually undetermined in that study. The chloroplast genome gives the most strongly supported indication of relationships among the three genera, but the possible concordance between plastid gene trees and species trees remains tentative, given that the chloroplast genome sequences of ~150 kbp still essentially represent a single-locus (linkage group) phylogeny (Ruhsam et al., 2015).

### Phylogenetic relationships within *Amana*

Within *Amana*, the rare species may be recently evolved ecotypes of the widespread *A. edulis*, quickly adapting lineage. As ecotypes, these rare species are expected to show no genetic distinction at neutral loci, and may not merit species recognition lineage (Oberle and Schaal, 2011). Nevertheless, the six *Amana* species exhibit sequence divergences in plastid genomes (Figure S2), ruling out the possibility of being ecotypes. Furthermore, our phylogenomic analyses as expected recovered *Amana* as a monophyletic genus (BS = 100%, PP = 1), and strongly supported its division into two clades: a widespread species (*A. edulis*) and a clade of five rare species (BS = 100%, PP = 1; Figure 5). The result indicates that although they are allopatrically distributed across East China/South Japan, the five rare species share a more recent common ancestor with each other than they do with *A. edulis*. Therefore, it is unlikely that any of these rare species originated from the widespread species through local geographical and ecological isolation by progenitor–derivative speciation (Crawford, 2010). In fact, the two sister lineages exhibit different eco-geographies: while *A. edulis* is widespread in lowland evergreen broad-leaved or temperate deciduous forests of East/North China, Japan, and the Korean peninsula, the rare species are endemic to the montane warm-temperate-deciduous (WTD) forest in East China/South Japan. In line with evidence from palaeomodeling of East Asian forest biomes (Harrison et al., 2001) and recent phylogeographic studies (reviewed in Qiu et al., 2011), the exceptionally high diversification rate in the “rare-species” clade is mainly driven by long-term allopatric population isolation (viz. vicariance) in which climate-induced eco-geographic isolation through (a)biotic displacement of WTD forested habitats at different spatial–temporal scales and over glacial and interglacial periods is the primary vicariance factor (see also Qiu et al., 2009). Overall, our phylogenomic analyses based on chloroplast genomes have provided the first successful attempt to clarify intrageneric relationships within *Amana*. However, based on distributional considerations, hybridization is still expected to occur between the widespread *A. edulis* and rare species within their zone of sympatry. Although these cp genome data have generated a fully resolved phylogeny of the genus *Amana* (Figure 5), it is not possible to use such data to classify hybridization events because cpDNA is generally uniparentally inherited (Birky, 1995). In the future, multi-locus phylogenies, phylogeography and palaeo-climatic niche modeling are required to explore the time scales and demographies of species divergences as well as hybridization in this genus.

## Author contributions

PL, YQ, and CF conceived the ideas; PL, RL, TO, and MC contributed to the sampling; MC performed the experiment; RL and WX analyzed the data. The manuscript was written by PL, RL, YQ, and KC.

### Conflict of interest statement

The authors declare that the research was conducted in the absence of any commercial or financial relationships that could be construed as a potential conflict of interest.

## Supplementary material

Figure S1 **Codon usage (gray bar) and relative synonymous codon usage (RSCU) value (red dot) of six *Amana* chloroplast genomes**.

Figure S2 **Sequence identity plots between five *Amana* chloroplast genomes, with *A. kuocangshanica* as a reference**. Annotated genes are displayed along the top. The vertical scale represents the percent identity between 50 and 100%. Genome regions are color coded as exon, intron, and conserved non-coding sequences (CNS).

Figure S3 **Repeat analyses in six *Amana* chloroplast genomes. (A)** Frequency of repeat types. **(B)** Frequency of repeats by length. **(C)** Summary of the shared repeats among species (ED, *A. edulis*; LA, *A*. *latifolia*; ER, *A*. *erythronioides*; AN, *A*. *anhuiensis*; KU, *A*. *kuocangshanica*; WA, *A*. *wanzhensis*).

Figure S4 **Simple sequence repeats (SSRs) in six *Amana* chloroplast genomes. (A)** Numbers of SSRs by length. **(B)** Distribution of SSR loci in the cp genomes. IGS, intergenic spacer region.

Table S1 **Codon usage and relative synonymous codon usage (RSCU) value of six *Amana* chloroplast genomes**.

Table S2 **Nucleotide variability (Pi) values and total number of mutation (Eta) in *Amana***.

Table S3 **Analyses of repeat sequences in six *Amana* chloroplast genomes**.

Table S4 **Simple sequence repeat (SSR) distribution in six *Amana* chloroplast genomes**.

Table S5 **Simple sequence repeat (SSR) polymorphism in six *Amana* chloroplast genomes**.
