# Supplementary figures and images for: Comparative Genomics and Phylogenomics of East Asian Tulips (Amana, Liliaceae)
Source: Front Plant Sci. 2017 Apr 4;8:451. doi: 10.3389/fpls.2017.00451 (PMC5378804; doi:10.3389/fpls.2017.00451)

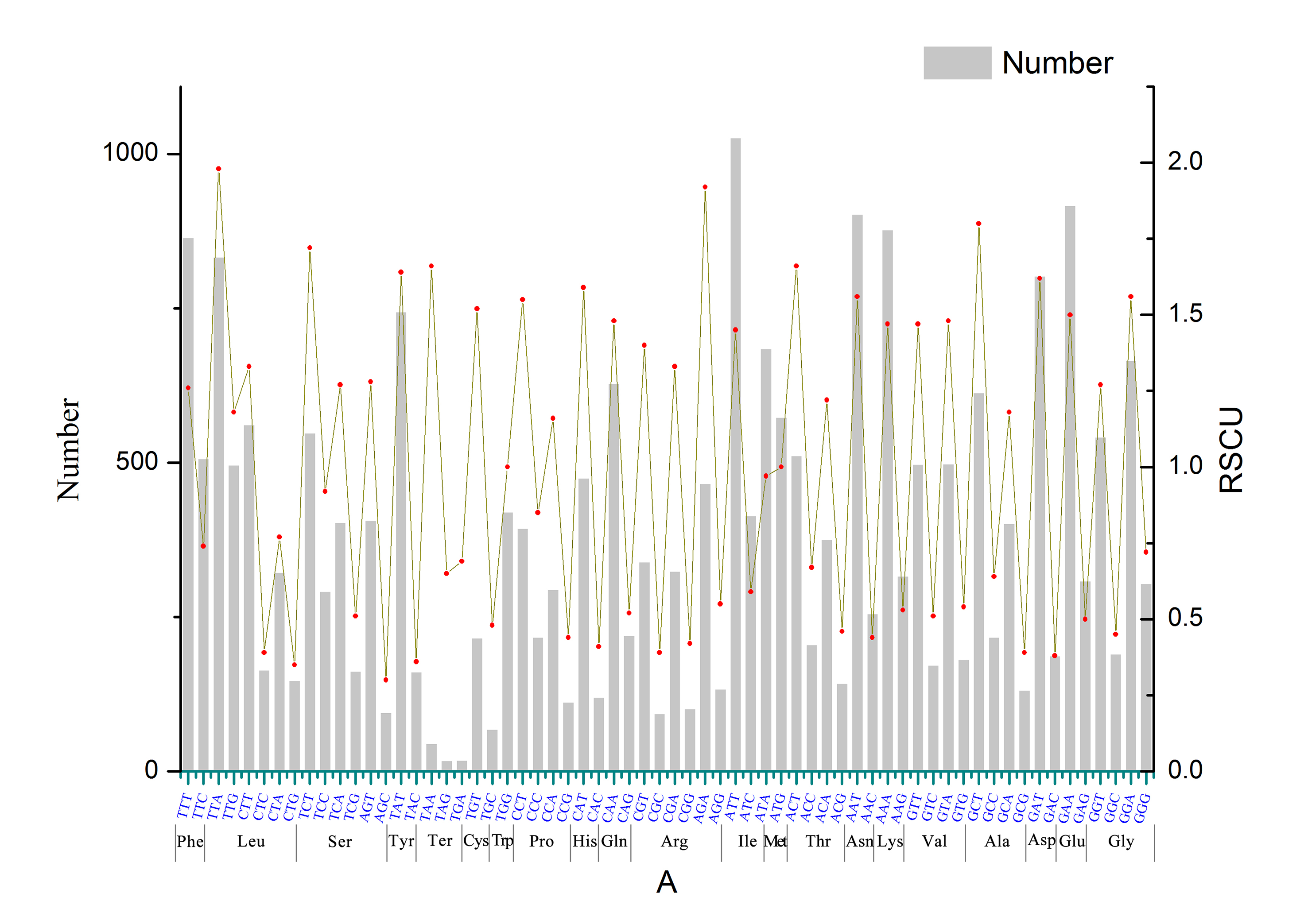

Supplement: Figure S1 — Codon usage (gray bar) and relative synonymous codon usage (RSCU) value (red dot) of six Amana chloroplast genomes. [file Image1.TIF]

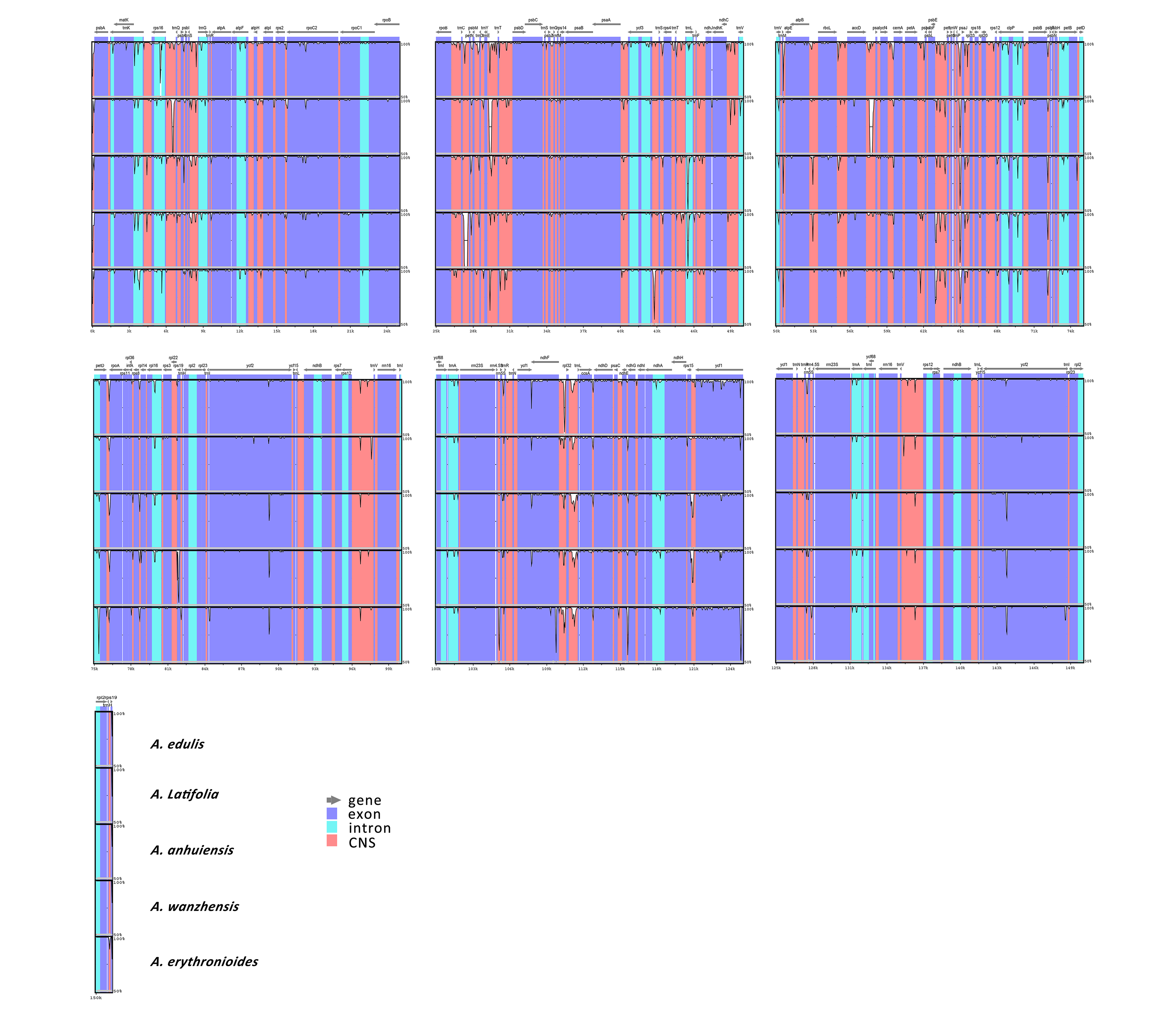

Supplement: Figure S2 — Sequence identity plots between five Amana chloroplast genomes, with A. kuocangshanica as a reference. Annotated genes are displayed along the top. The vertical scale represents the percent identity between 50 and 100%. Genome regions are color coded as exon, intron, and conserved non-coding sequences (CNS). [file Image2.TIF]

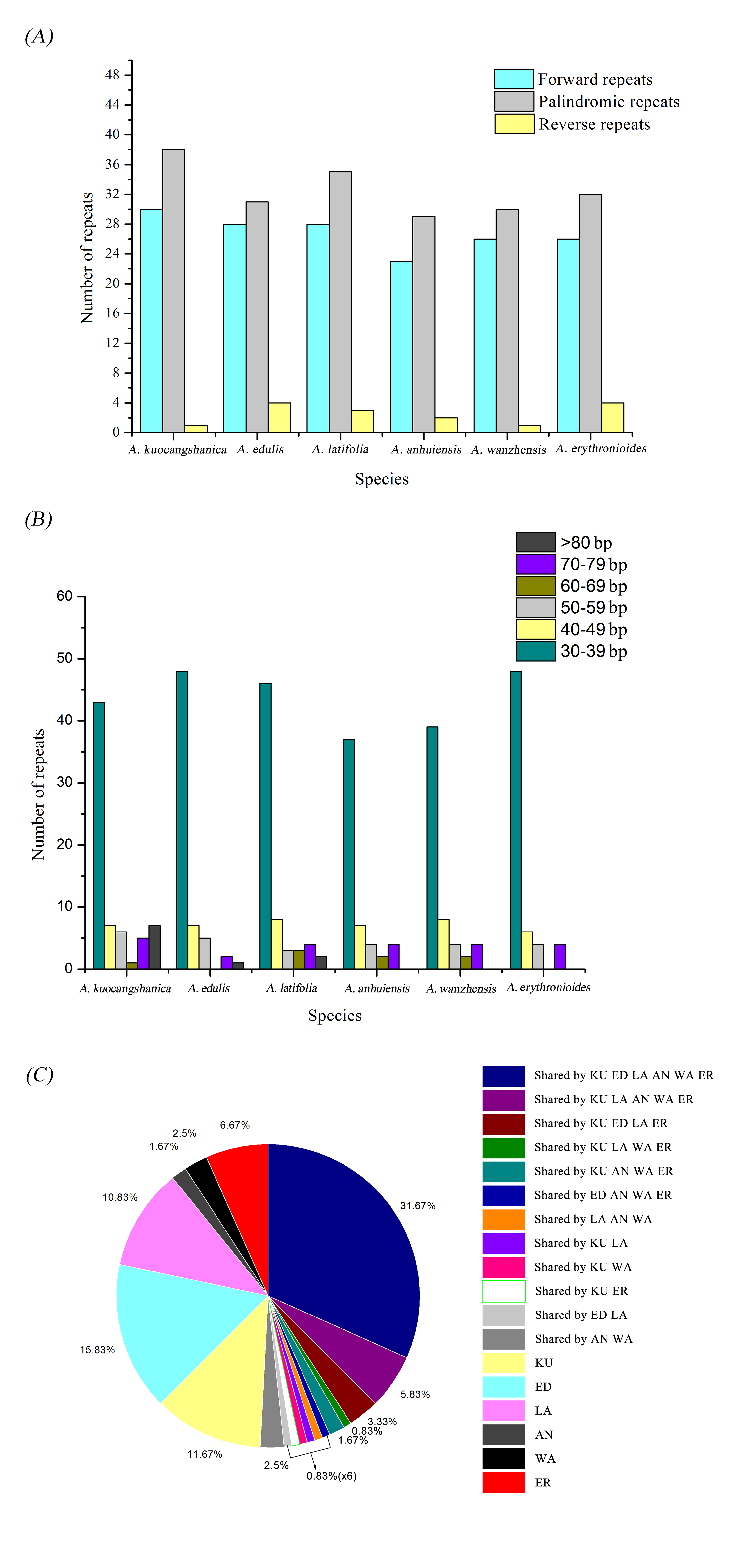

Supplement: Figure S3 — Repeat analyses in six Amana chloroplast genomes. (A) Frequency of repeat types. (B) Frequency of repeats by length. (C) Summary of the shared repeats among species (ED, A. edulis; LA, A. latifolia; ER, A. erythronioides; AN, A. anhuiensis; KU, A. kuocangshanica; WA, A. wanzhensis). [file Image3.TIF]

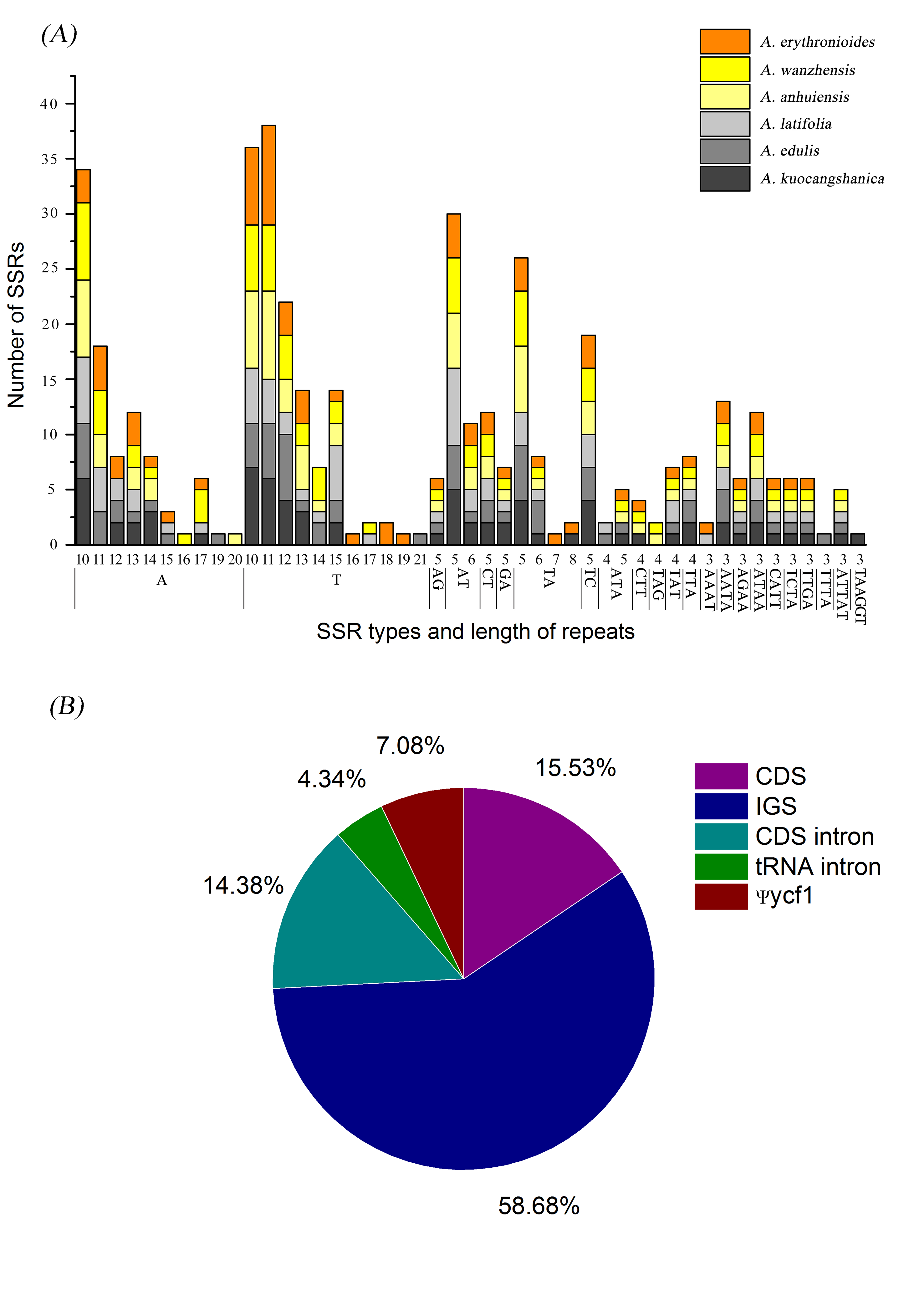

Supplement: Figure S4 — Simple sequence repeats (SSRs) in six Amana chloroplast genomes. (A) Numbers of SSRs by length. (B) Distribution of SSR loci in the cp genomes. IGS, intergenic spacer region. [file Image4.TIF]
